# Biodegradable collagen matrix implant versus mitomycin-C in trabeculectomy: five-year follow-up

**DOI:** 10.1186/s12886-016-0198-0

**Published:** 2016-03-05

**Authors:** Salvatore Cillino, Alessandra Casuccio, Francesco Di Pace, Carlo Cagini, Lucia Lee Ferraro, Giovanni Cillino

**Affiliations:** Department of Experimental Biomedicine and Clinical Neuroscience, Ophthalmology Section, University of Palermo (Italy), via Liborio Giuffrè, 13, 90127 Palermo, Italy; Department of Sciences for Health Promotion and Mother-Child Care “G. D’Alessandro”, University of Palermo, Via del Vespro 127, I, 90127 Palermo, Italy; Department of Surgical and Biomedical Sciences, Section of Ophthalmology, University of Perugia, Piazza Menghini 1. S. Andrea delle Fratte, 06156 Perugia, Italy

**Keywords:** Mitomycin-C, Ologen, Trabeculectomy, Extended 5-yrs follow-up

## Abstract

**Background:**

Clinical studies comparing trabeculectomy augmented with Ologen implant (OLO) versus trabeculectomy plus mitomycin-C (MMC) show contradictory results. To obtain long-term data, we report an extended 5-year follow-up trial evaluating the safety and efficacy of OLO as adjuvant compared to low-dosage MMC in trabeculectomy.

**Methods:**

Forty glaucoma patients (40 eyes) assigned to trabeculectomy with MMC or Ologen. Primary outcome: target IOP at ≤21, ≤17 and ≤15 mmHg; complete and qualified success endpoint rates. Secondary outcomes: visual acuity (VA), mean deviation (MD), bleb evaluation, according to Moorfields Bleb Grading System (MBGS); spectral domain OCT (SD-OCT) bleb examination; number of glaucoma medications; frequency of postoperative complications.

**Results:**

The mean preoperative IOP was 26.7(±5.2) in MMC and 27.3(±6.0) in OLO eyes. Mean 60-month percentage reduction in IOP was significant in both groups [40.9 (±14.2) and 42.1(±13.3) *P* = 0.01], with an endpoint value of 15.2 (±3.2) and 15.8 (±2.3) mmHg in MMC and OLO, respectively. Complete success rates at ≤ 21 mmHg target IOP were 65 % and 70 %, at ≤17 mm Hg 60 % and 55 %, and at the ≤15 mm Hg target IOP 35 % and 45 % in MMC and OLO, respectively.

The Kaplan–Meier curves did not differ both for complete and qualified success at any target IOP, with no significant endpoint intergroup difference at ≤ 15 mm Hg (log-rank *P* = 0.595).The intergroup MBGS scores differed due to reduced central and peripheral vascularity in MMC group (P = 0.027; *P* = 0.041).

SD-OCT analysis denied differences in bleb height between MMC vs OLO (140.5 ± 20.3 μ vs 129.2 ± 19.3 μ respectively; *P* =0.079).

Mean antiglaucoma medications were significantly reduced (*P* < 0.0005) from 2.5 (±0.3) to 1.2 (±0.4) in MMC and from 2.6 (±0.2) to 1.4 (±0.3) in OLO group, with no intergroup differences (*P* = 0.08).

Six (30 %) cystic thin avascular blebs without oozing were recorded in the MMC group and 2 (10 %) in the OLO group, without intergroup difference (*P* = 0.235).

**Conclusions:**

Our extended follow-up results confirm that Ologen implant yields efficacy and long-term success rates quite similar to MMC, with at least equivalent safety.

**Electronic supplementary material:**

The online version of this article (doi:10.1186/s12886-016-0198-0) contains supplementary material, which is available to authorized users.

## Background

Trabeculectomy with mitomycin-C (MMC) today is still regarded as the gold-standard in glaucoma surgery. Yet, in many studies MMC-related complications such as prolonged wound leaks, hypotony with choroidal effusions and maculopathy, thin avascular blebs, and/or bleb leaks with late infection are frequently reported [1–9].

A biodegradable collagen-glycosaminoglycan copolymer matrix implant (Ologen®) has been proposed as an alternative adjuvant, used as a spacer to mechanically separate the sub conjunctival and episcleral tissues to preventing fibrosis, and also helps in reorganizing the subconjunctival scar formation.

In fact, it should induce fibroblasts and myofibroblasts to grow randomly into its porous structure and secrete a loose connectival matrix, reducing the scarring degree. The implant is recommended to be placed subconjunctivally over the scleral flap posteriorly and possibly a small portion covering the scleral flap, else the ologen disc would act as a mechanical tamponade and prevent fluid outflow from the sub scleral space.

In 2010, a medium-term RCT did not show any intraocular pressure-lowering advantage of the Ologen-augmented trabeculectomy vs trabeculectomy alone, with a higher yet not significant incidence of complications with the collagen implant [10]. In the same year another randomized study of MMC-augmented trabeculectomy vs trabeculectomy using Ologen showed a lower complete success rate but a lower bleb-associated complication rate in Ologen group [11].

In 2011, we published the results of a 24-month, randomized prospective clinical trial on Ologen implant vs MMC in trabeculectomy [12]. The intraocular pressure (IOP) reduction was significant at endpoint in all groups (*P* = 0.01). The rates and Kaplan–Meier curves did not differ for both complete and qualified success at any target IOP. The bleb height in the Ologen-treated group was higher than in the MMC one (*P* < 0.05). No adverse reaction to Ologen was noted.

In the past three years, a number of clinical studies have compared the efficacy and tolerability of trabeculectomy augmented with Ologen versus trabeculectomy plus MMC, with somehow contradictory results [13–20].

To obtain more data on the long-term IOP lowering effect of the Ologen implant compared to MMC as adjuvant in trabeculectomy, we extended to five years the follow-up on the same cohort of subjects of our abovesaid study. The parameters measured included IOP, bleb morphology, and frequency of complications.

## Methods

This study is an extended, 60-month follow-up data. The protocol of the randomized study had been approved by the Ethical Committee of the University Hospital of Palermo (Italy) on December 2007 and registered under the number 08/07 (12 September 2007) .We reviewed the records of 40 patients who had been randomly assigned to undergo a trabeculectomy with MMC (MMC group) or a trabeculectomy with Ologen implant (OLO group) for primary open-angle (POAG) or pseudoexfoliative glaucoma (PEXG) at the Department of Ophthalmology of the University of Palermo. The data used to generate the 5-year life table analysis were collected over the interval between enrollment in the study and 60 months following surgery.

In the previous prospective randomized clinical trial [12] a sample size of 40 patients (20 eyes in each group) had been chosen to achieve a power of 90 % for detecting a 3-mmHg difference in IOP between treatment procedures, assuming a standard deviation of three mmHg and a two-sided α error of 5 %.

In accord with tenets of Declaration of Helsinki a written informed consent has been obtained from all patients, also covering approval to publish images. We screened for uncontrolled glaucoma 65 consecutive Caucasian patients at the Glaucoma Center of the Department of Ophthalmology between January and December 2008. Sealed envelope technique from surgical chart number was used to ensure randomization just before surgery. Skilled ophthalmologists and optometrists masked to randomization collected the clinical data and the outcomes. Inclusion criteria were age 18 or older, diagnosis of POAG or PEXG with mild to moderate visual field damage [21], IOP above 21 mm Hg or visual field deterioration on maximum-tolerated medical therapy.

Exclusion criteria were advanced glaucoma or split fixation on visual field, normal-tension glaucoma, use of medications for acute or chronic disease that could affect the outcomes (eg, immunodeficiency, connective tissue disease, and diabetes), clinically significant cataract, previous ocular trauma or surgery. Included preoperative data consisted of age, gender, type of glaucoma, type and number of antiglaucoma medications; the ophthalmic examination included Goldmann applanation tonometry, biomicroscopy; Snellen visual acuity and computerized Humphrey visual field testing (Humphrey Visual Field Analyzer; HFA; Carl Zeiss Meditec. Inc.).

The primary outcome was IOP evaluated at three different IOP target levels: ≤ 21, ≤17, and ≤15 mmHg. Complete and qualified success were defined as usual, i.e. without medications in the first case and regardless of medications in the second one. Secondary outcome measures included visual acuity (VA), mean deviation (MD) change by Humphrey 24-2 full threshold testing, bleb evaluation according to Moorfields Bleb Grading System (MBGS), and bleb SD-OCT analysis. Number of glaucoma medications and frequency of postoperative adjunctive procedures and complications were also evaluated.

### Surgical technique and follow-up

The technique has been described in detail in the previous study [12]. All operations, carried out by one experienced surgeon (SC), included a superior fornix-based conjunctival/tenons flap, with a rectangular scleral flap at the 12-o’clock position, fashioned according to the ‘Moorfields Safer Surgery System’ procedure [22, 23]. When MMC (Kyowa S.r.l., Milan, Italy) was the randomized adjunctive therapy, Weck-cell sponge pieces soaked with 0.2 mg/ml MMC were simultaneously placed under the dissected conjunctiva/tenon surrounding the scleral flap [22, 23], and on the scleral bed, underneath the scleral flap, for a total time of 2 min [24]. After thorough irrigation, trabeculectomy was performed with a Crozafon-De Laage punch. A peripheral iridectomy was performed, the anterior chamber was filled with viscoelastic device, and the scleral flap was closed with two minimally tensed 10-0 nylon sutures -one at each corner- in MMC cases and with one long and loose stitch in OLO cases. A cylindrical Ologen implant (model number 830601, Aeon Astron Europe BV), 2.0 ± 0.3 mm in height x 6.0 ± 0.5 mm in diameter was then centered over the top of scleral flap and underneath the conjunctiva in the latter leaving the limbal end of the stitch partially uncovered. The implants used in this cohort of patients consisted of porcine based lyophilized cross-linked type I atelocollagen (≥90 %) and glycosaminoglycans (≤10 %). Degradation time of this type was around 180 days. A newer version with exactly the same composition but with a bit shorter degradation time is now available.

The conjunctival flap was secured to the limbus with a tight 10-0 nylon running suture with buried knots.

Postoperatively, all eyes were treated with tapering doses of topical tobramycin 0.3 % and dexamethasone drops 0.1 % for 2 months. The ‘intensified postoperative care’ (IPC) protocol [25] was followed in all cases and included more frequent topical steroid administration if corkscrew bleb vessels were present. Instillation of 1 % atropine drops during the first few days was based on the hypotony degree. Adjunctive procedures included the Carlo Traverso maneuver [26], laser suture lysis or bleb needling of encapsulated blebs (without antimetabolites).

If postoperative IOP measurements were >21 mmHg after topical steroid withdrawal, IOP-lowering medication was added.

When, during the extended follow-up, a patient developed a clinically significant cataract in the operated eye, a standard sutureless cataract surgery was performed through a temporal 2.5-mm near-clear corneal tunnel incision with a precalibrated knife (Clearcut, Alcon Italia S.P.A., Milan, Italy). Phacoemulsification was performed with the Alcon Infiniti Vision System (Alcon Italia S.P.A.), using the Ozil torsional handpiece in the majority of cases, avoiding any contact with the bleb area. The implantation of acrylic hydrophobic foldable intraocular lenses (IOLs) was performed using an Unfolder Emerald injector system (AMO Italy, Rome, Italy) or a Monarch II injector (Alcon Italia S.P.A.). The surgical wound was closed by stromal hydration. All patients received topical ofloxacin (Exocin, Allergan SpA, Rome, Italy) for 3 days preoperatively and tobramycin and dexamethasone ophthalmic suspension (Tobradex, Alcon Italia S.P.A.) for one week postoperatively, followed by nepafenac ophthalmic suspension (Nevanac, Alcon Italia S.P.A.) or bromfenac ophthalmic solution (Yellox, Bausch Lomb, Italia), for 3 weeks. In case of topical antiglaucomatous therapy use, this was continued even throughout the cataract surgery period. Even in cataract surgery cases, if postoperative IOP measurements were >21 mmHg after topical steroid withdrawal, IOP-lowering medication was added.

Expanded follow-up implied six-monthly examinations till the 60^th^ month. All patients were thoroughly informed about the importance of a periodical examination, and were regularly visited by the same staff to create a relationship of empathy. We were lucky to be able to follow all patients for 5 years. We report data collected at 36, 48 and 60-month observation times (Additional file 1).

Follow-up visits included assessment of VA, IOP, biomicroscopic findings, number of antiglaucomatous medications, and postoperative complications. Signs of inflammation, such as cells and flare, were graded from 0 to 4. The MBGS [27] -using recorded photographs- was used for bleb grading by a single observer (GC) at each follow-up visit. Cystic or avascular blebs were noted.

Spectral domain optical coherence tomography (SD-OCT; Topcon 3DOCT-1000, Topcon Corporation, Tokyo, Japan), already performed at the previous study end point (24 month), was repeated at the 60-month observation time for bleb evaluation by ophthalmologists masked to clinical data (CG and LLF). A bleb was identified as successful or failed based on the presence or absence of bleb wall thickening and microcystic or hyporeflective intrableb wall structures (with respect to a ≤17 mmHg target IOP level [28, 29]). Thereafter, we add measurements of some bleb parameters, performed with calipers using the device’s built-in software in a masked fashion. Bleb wall thickness was defined as the distance between the first reflective signal from the conjunctiva to the top of the sub-Tenon fluid space. Because the bleb wall thickness may vary along the scan, we analyzed only the maximum and minimum distances [30].

The whole bleb height was defined as the maximum vertical length from the outer margin of the bleb wall and the highly reflective margin of the scleral surface in the cross-sectional image.

### Statistical analysis

Statistical analysis of quantitative and qualitative data, including descriptive statistics, was performed for all items. The independent Student t-test and the Mann–Whitney U statistic test were used for parametric and non-parametric analysis, respectively. Discrete variables were analyzed using the chi square test and Fisher exact test, as needed. Intragroup parametric and non-parametric analysis were carried on by using the paired-samples Student t-test and paired Wilcoxon signed-rank test respectively. Success was evaluated on the basis of Kaplan–Meier cumulative probability (log-rank test). To assess intraobserver reproducibility and consistency, an internal quality control system was established before the study onset by using three consecutive independent interpretations of the same SD-OCT scan, together with the unweighted Cohen kappa (k) test [31]. Data were analyzed by the Epi Info software (version 6.0, Centers for Disease Control and Prevention, Atlanta, GA, USA) and IBM SPSS Software 21.0 version (SPSS, Inc., Chicago, Ill, US). All *P*-values were two-sided and *P*-values less than 0.05 were considered statistically significant.

## Results

Patients in the two treatment groups did not significantly differ in any of the preoperative parameters. Two women with early-onset glaucoma, aged 36 and 38 years, were included, the former in the MMC group and the latter in the OLO one.

As above said, all 40 patients completed the 60-month follow-up. During the 3^rd^ to 4^th^ year of follow-up, 3 cases (15 %) in the MMC group and 2 cases (10 %) in the OLO group developed a clinically significant cataract with VA decrease in the operated eye, and therefore underwent cataract surgery with IOL implantation (Table 1).

**Table 1:** Preoperative characteristics of patients who underwent trabeculectomy, Snellen acuity and MD change, and 3^rd^ to 4^th^ year follow-up cataract surgery cases

	MMC group	OLO group	*P*
Gender (M/F), N°	11/9	12/8	1.0^a^
Age, yrs. (mean ± SD)	63.2(7.2)	65.8(6.4)	0.234^b^
Right/left eyes, N°	7/13	11/9	0.340^a^
Type of glaucoma (POAG/PEXG), N°	12/8	13/7	1.0^a^
Preoperative IOP, mmHg (mean ± SD)	26.7(5.2)	27.3(6.0)	0.736^b^
Baseline CDVA, decimal notation	0.8 (±0.33)	0.75 (±0.31)	0.624
Endpoint CDVA, decimal notation	0.8 (±0.40)	0.8 (±0.35)	1.0
Baseline MD, dB (mean ± SD)	-7.80(4.57)	-7.41(5.35)	0.805^b^
Endpoint MD, dB (mean ± SD)	-7.60(4.3)	-7.50(5.6)	0.949^b^
Preoperative medications, N° (mean ± SD)	2.5(0.3)	2.6(0.2)	0.222^b^
Duration of preoperative antiglaucoma therapy, yrs. (mean ± SD)	5.7(1.8)	6.3(1.4)	0.246^b^
Cataract surgery cases	3 (15 %)	2 (10 %)	1.0

^a^Chi square test or Fisher exact test, as needed; ^b^independent Student t test;

MMC = Mitomycin-C; OLO = Ologen; SD = standard deviation;

POAG = primary open angle glaucoma; PEXG = pseudoexfoliation glaucoma; CDVA = Corrected Distance Visual Acuity; MD = Humphrey Visual Field Analyzer Mean deviation.

**Fig. 1:**
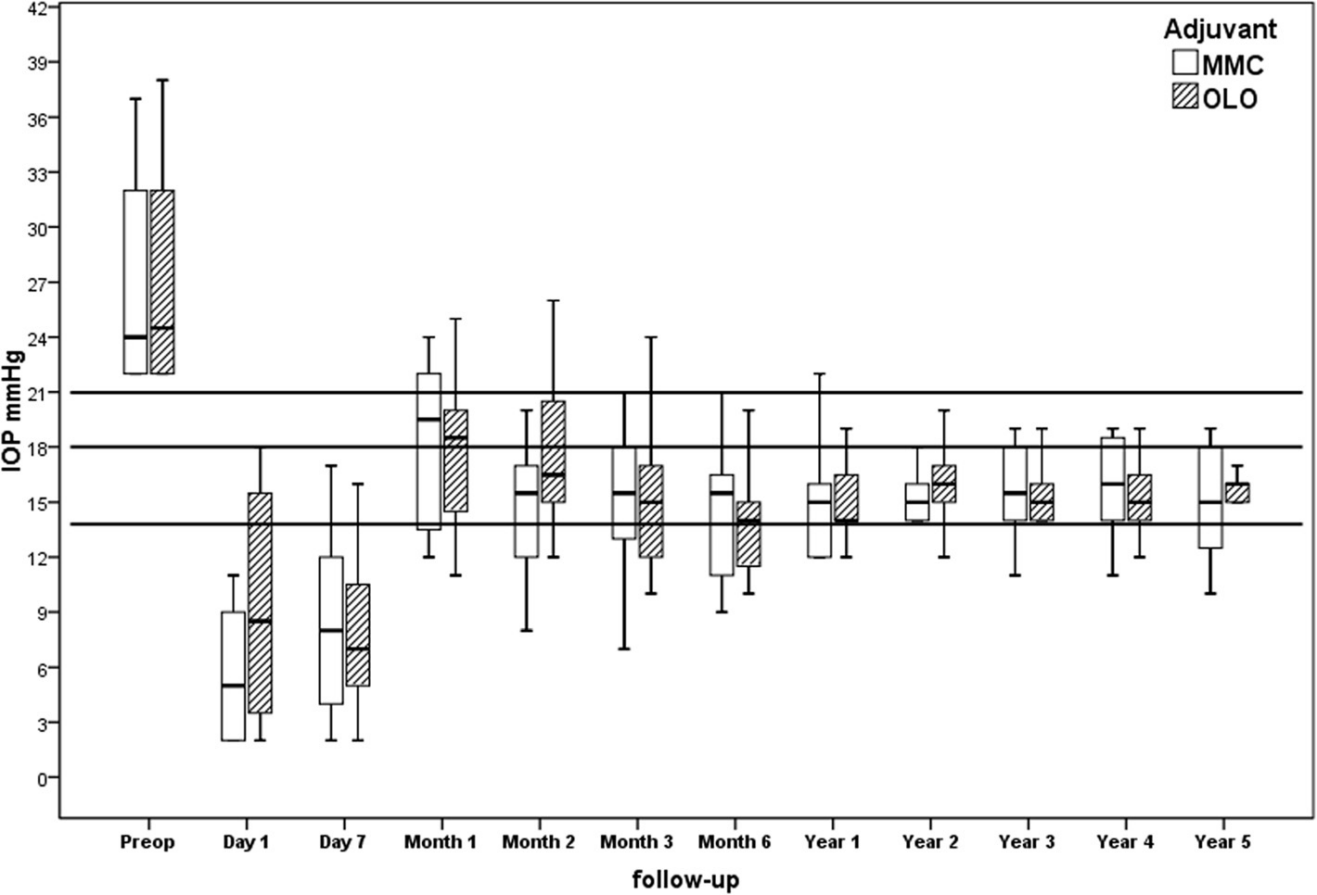
Box-plot representation of IOP values over 60-months follow-up: median values (dark lines), error standard (T-bars)

Mean Snellen acuity and visual field test MD at the 5-years end point did not differ from the baseline in both groups (Table 1). The mean preoperative IOP (±SD) was 26.5 (±5.2) in MMC eyes and 27.3 (±6.0) in OLO eyes, without significant intergroup difference. One-day postoperatively, the IOP dropped to 5.2 (±3.5) and 9.2 (±5.5) mmHg, respectively (*P* = 0.009). No intergroup difference was present at any scheduled postoperative time interval. The postoperative IOP reduction was significant at the 24-month endpoint in both groups (*P* = 0.01). At the extended 36-month time interval, the mean IOP was 15.6 (±2.6) in the MMC group and 15.9 (±2.5) mmHg in the OLO group (*P* = 0.706), while at the 48^th^ month the IOP values were 15.9 (±2.6) and 15.3 (±3.4) respectively (*P* = 0.563) Finally, at the 60^th^ month the IOP values were 15.2 (±3.2) and 15.8 (±2.3) respectively (*P* = 0.579). The endpoint percentage IOP reduction from baseline was 40.9 (±14.2) and 42.1(±13.3), respectively (*P* = 0.827) (Table 2 and Fig. 1). The two cases of early-onset glaucoma exhibited an IOP within the low teens without medications.

**Table 2:** Postoperative IOP (mmHg) in the surgical groups. Mean (±SD; 95%CI); % change in IOP from baseline

	MMC group	OLO group	*P* ^a^
3^rd^ month	14.7(3.9; 12.9-16.4)	15.0(3.8; 13.3-16.7)	0.806
	44.5 %	45.1 %	
6^th^ month	14.7(4.3; 12.7-16.6)	14.1(3.1; 12.6-15.4)	0.615
	44.5 %	48.4 %	
12^th^ month	15.0(3.0; 13.6-16.4)	15.2(2.8; 13.8-16.4)	0.828
	43.4 %	44.3 %	
24^th^ month	16.0(2.9; 14.6-17.4)	16.5(2.1; 15.5-17.4)	0.536
	39.6 %	39.5 %	
36^th^ month	15.6 (2.6; 14.3-16.9)	15.9 (2.5; 14.6-17.2)	0.706
	39.8 %	40.2 %	
48^th^ month	15.9 (2.6; 14.5-17.2)	15.3 (3.4; 13.4-17.1)	0.563
	38.8 %	42.3 %	
60^th^ month	15.2 (3.2; 13.4-16.9)	15.8 (±2.3; 14.4-17.1)	0.579
	41.0 %	42.1 %	

^a^ Independent Student t test; MMC = Mitomycin-C; OLO = Ologen; SD = standard deviation

In the cases who underwent cataract phacoemulsification, the mean pre-operative IOP was 16.9 (±2.3) in MMC eyes and 17.0 (±2.9) in OLO ones, whilst the endpoint IOP was 17.2 (±2.5) and 16.5 (±2.7) respectively (*P* = 0.885, and *P* = 0.875, respectively). The number of antiglaucoma medications, 0.7 (0.6) in MMC and 0.5 (0.7) in OLO cases did no change after cataract surgery. All cataract patients experienced a post-operative VA increase of at least 2 lines.

Table 3 reports the success rates in the study groups. At the 60-month endpoint follow-up, the values regarding complete success at ≤ 21 mmHg target IOP were 65 % and 70 % respectively for the MMC and the OLO group, with a qualified success of over 85 % in both. At the ≤17 mm Hg target IOP, complete success percentages were 60 % and 55 %, and qualified ones 70 % and 75 % respectively. At the ≤15 mm Hg target IOP level complete success was recorded in 35 % and 45 % of cases, and qualified one in 40 % and 50 % of patients respectively with no significant difference at any follow-up time.

**Table 3:** Success rates (%) at the 60-month follow-up study endpoint in the surgical groups at three target IOP levels

	MMC group	OLO group	*P* ^a^
≤21 mmHg			
Complete success	13(65 %)	14(70 %)	1.0
Qualified success	17(85 %)	18(90 %)	1.0
≤17 mmHg			
Complete success	12(60 %)	11(55 %)	1.0
Qualified success	14(70 %)	15(75 %)	1.0
≤15 mmHg			
Complete success	7(35 %)	9(45 %)	0.747
Qualified success	8(40 %)	10(50 %)	0.751

^a^Fisher exact test; MMC = Mitomycin-C; OLO = Ologen

**Fig. 2:**
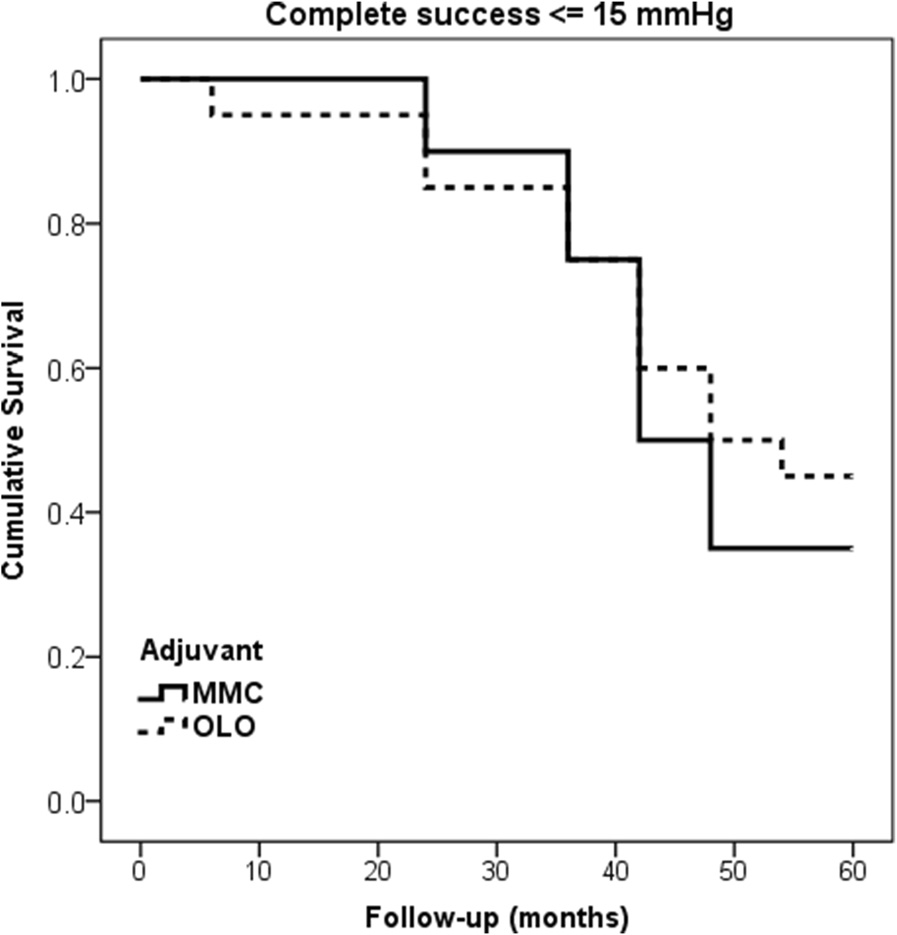
Kaplan-Meier cumulative probability curve of complete success (without medications) at ≤15 mmHg target IOP in MMC (solid line) vs OLO group (dotted line) (log-rank P = 0.595) at 60 month follow-up. MMC = Mitomycin-C; OLO = Ologen

At 24-month follow-up, the Kaplan–Meier cumulative survival curves relating either the ≤21, ≤17, or ≤15 mmHg target IOP had not showed significant intergroup differences for complete or qualified success rates. Figure 2, indicates the same parameter behavior relating complete success rates at ≤ 15 mm Hg target IOP up to the 60^th^ month end point, with no significant intergroup difference (log-rank *P* = 0.595).

The area, height and vascularity MBGS scores did not generally differ on an intragroup and intergroup basis, maintaining stability till the 24^th^ month. One exception was height, whose mean score was higher in OLO group at the third month (2.0 ± 0.8 vs 1.3 ± 0.7; *P* = 0.009; Mann–Whitney U statistic test), maintaining a higher yet not significant value till the first 24th month end point.

**Fig. 3:**
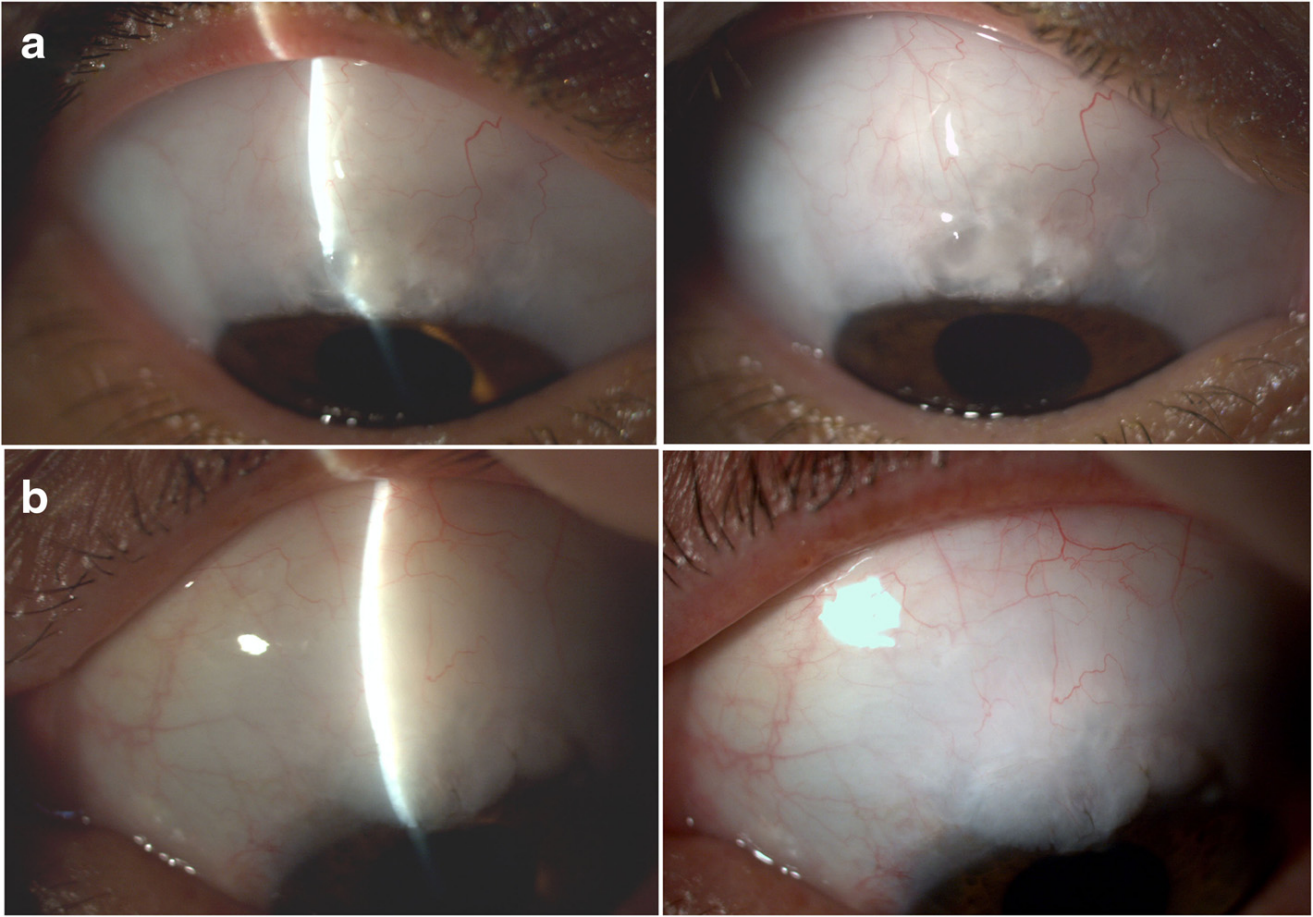
Slit light and diffuser photographs of two cases. Patient **a** (top): 38 yrs-old woman from MMC group. **a** diffuse bleb with central avascular/cystic area can be seen. Patient **b** (bottom): 52 yrs-old man from OLO group. A diffuse bleb with almost normal vascularity is present

No significant difference was found between the 24-month and the 60-month values, either with respect to central area (*P* = 0.729 in MMC and *P* = 0.231 in OLO group), maximal area (*P* = 0.769 and *P* = 0.395, respectively) and height (*P* = 0.408 and *P* = 0.478, respectively). The 60-month mean MBGS score values in MMC vs OLO group were 2.7 ± 0.7 vs 2.3 ± 0.9 (*P* = 0.191) relating central area, 2.9 ± 0.8 vs 2.6 ± 1.0 (*P* = 0.378) relating maximal area, and 1.2 ± 0.5 vs 1.2 ± 0.8 (*P* = 0.722) relating height, without intergroup difference. At the same time interval, central, peripheral, and non-bleb vascularity mean score values in MMC vs OLO group were 0.6 ± 0.3 vs 0.9 ± 0.5, 1.3 ± 0.3 vs 1.1 ± 0.3, and 1.6 ± 1.0 vs 1.4 ± 1.0, with intergroup difference (*P* = 0.027; *P* = 0.041; *P* = 0.531), respectively, relating the central and peripheral score. Figure 3 shows two cases with diffuse bleb, one from MMC and the other from OLO group, with a central avascular/cystic area in the former and almost normal vascularity in the latter.

There was high intraobserver reproducibility for SD-OCT analysis (k = 0.7403, 95 % CI: 0.70–0.86). Table 4 reports the successful bleb frequencies, with respect to a ≤17 mmHg target IOP level, at 5-year follow-up.

**Table 4:** 60th month bleb success rates(%) at ≤17 mmHg target IOP in the surgical groups according to the SD-OCT analysis

	MMC group 60th mo	OLO group 60th mo	P 60th mo
Successful bleb / eyes with complete success	10/12	9/11	1.0^a^
Failed bleb / eyes without complete success	6/8	7/9	1.0^a^
SD-OCT sensitivity	83 %	82 %	0.627^b^
SD-OCT specificity	75 %	78 %	0.669^b^

^a^Fisher exact test; ^b^Chi-square test for the comparison of two proportions

(from independent samples), expressed as a percentage;

MMC = Mitomycin-C; OLO = Ologen

**Fig. 4:**
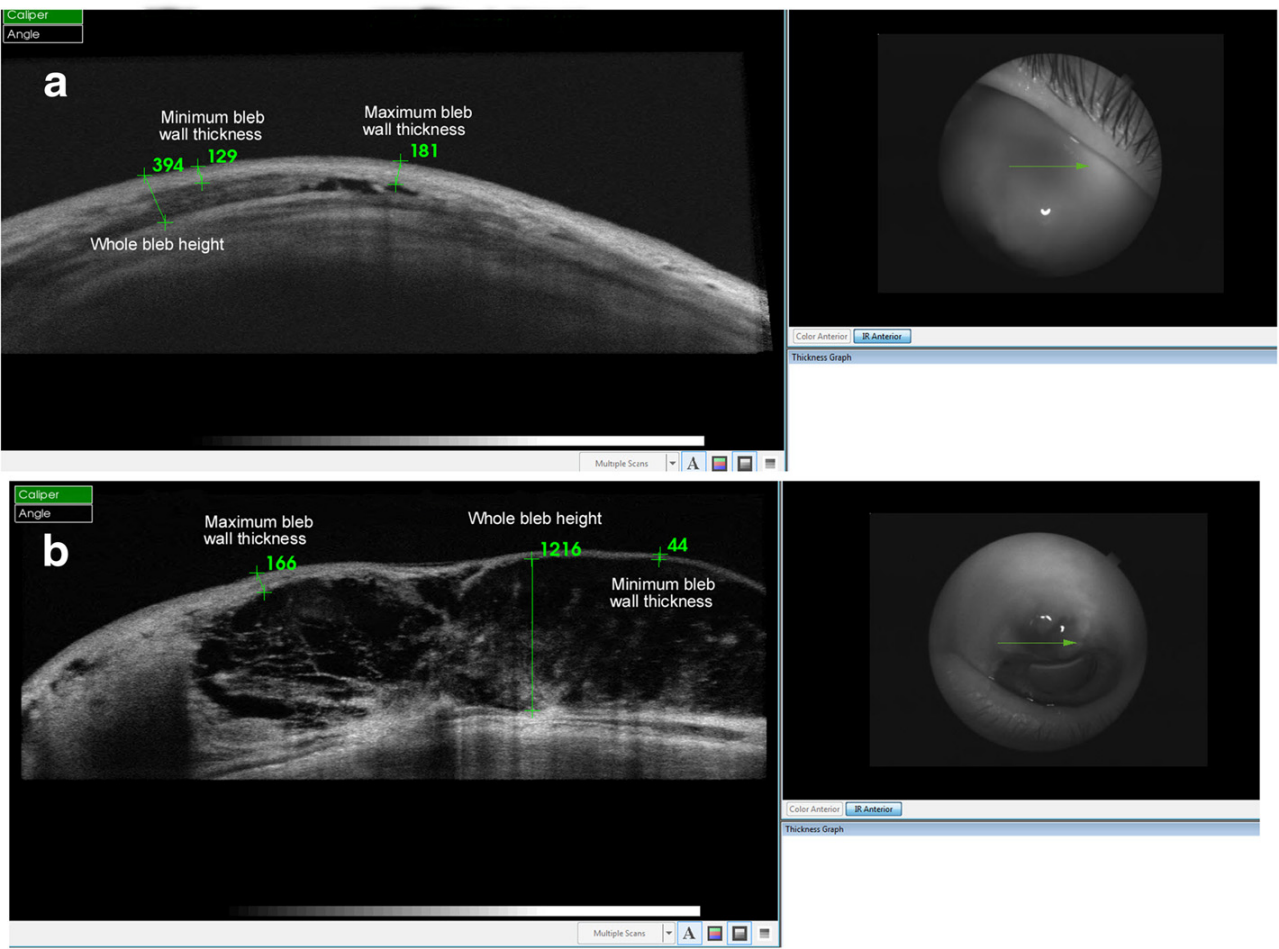
SD-OCT imaging of blebs at the 60-month end point with complete success based on ≤ 15 mmHg target IOP. (**a**): Thick bleb from OLO group. (**b**): Thin bleb from MMC group. The bleb (**a**), from the OLO group, exhibited a more diffuse pattern

Figure 4 displays examples of bleb thickness parameters obtained using the SD-OCT built-in software at the 60-month end point. Eight eyes, 6 in the MMC and 2 in the OLO group, with a cystic thin bleb, exhibited minimum bleb wall thickness mean values significantly smaller (54.5 ± 14.9 μ) than the remaining eyes (134.2 ± 17.3 μ) (*P* = 0.004), in agreement with the clinical appearance. No differences were found in terms of whole bleb height between groups (140.5 ± 20.3 μ vs 129.2 ± 19.3 μ respectively; *P* = 0.079).

The mean number of antiglaucoma medications was significantly reduced at end point in both groups (*P* < 0.0005 in both cases): from 2.5 (±0.3) to 1.2 (±0.4) and from 2.6 (±0.2) to 1.4 (±0.3) in the MMC and OLO groups, respectively, without significant intergroup differences (*P* = 0.08).

The Carlo Traverso maneuver was employed in two patients in each group between the 1st and the 14th postoperative day. Four cases (20 %) in the MMC group and three cases (15 %) in the OLO group -without intergroup difference- underwent Laser suture lysis between the first and the second postoperative week. Bleb needling was performed from one to four times in seven (35 %) and six (30 %) patients respectively, again without intergroup difference.

The frequency of early postoperative complication did not significantly differ between the two groups, even if a tendency toward more frequent early bleb leakage was noted in the OLO group, and toward more frequent early hypotony, defined as an intraocular pressure (IOP) equal to or less than 6 mmHg at the first postoperative day, and choroidal detachment in MMC one. No adverse reaction to the Ologen implant, matrix extrusion, or conjunctival erosion was present in OLO group. At the 60-month endpoint, three more cases of clinically significant cataract with VA decrease, 2 in the MMC and 1 in the OLO group, were found, to a total of 5 and 3 cases respectively during the whole follow-up. Five (25 %) patients in the MMC group and 4 (20 %) in the OLO one experienced > 1 line VA loss due to cataract or to age-related macular degeneration (AMD). Six (30 %) cystic thin avascular blebs without oozing, as above said, were recorded in the MMC group and 2 (10 %) in the OLO group, without intergroup difference (*P* = 0.235) (Table 5).

**Table 5:** Frequency (%) of 60-month postoperative complications in the surgical groups

	MMC group	OLO group	*P* ^a^
Cataract	5(25 %)	3(15 %)	0.694
Age-related macular degeneration	2(10 %)	2(10 %)	1.0
Loss > 1 line VA	5(25 %)	4(20 %)	1.0
Thin avascular bleb	6(30 %)	2(10 %)	0.235

^a^Fisher exact test; MMC = Mitomycin-C; OLO = Ologen

## Discussion

Both early and long-term complications are still reported in MMC-augmented trabeculectomy: As pointed out in our previous study [12], factors such as flaps fashioning, suturing technique and prolonged fibroblast inhibition -with thin bleb leaking- are responsible for these problems [8, 9, 22, 23, 32].

In our previous study, the early postoperative hypotony rate is really quite high in both groups, probably due to the loose stitches without fashioning releasable sutures: the latter technique could avoid this complication [12]. Anyway, the reduced tendency to first postoperative day’s hypotony in the OLO cases, when compared with the MMC ones, can be explained as a mechanical Ologen-induced aqueous outflow modulation.

The extended 5-year results indicate that the postoperative IOP behavior is quite similar in both groups, with a highly significant and stable IOP reduction, stable VA and MD, and reduced administration of antiglaucoma medications throughout the 60-month follow-up, without intergroup differences. The equivalence in efficacy of OLO vs MMC is further confirmed by their endpoint success rates at the three different target IOP levels. Even if it is well known that phacoemulsification leads to an increased risk of bleb failure of approximately 33 %, with changes in bleb morphology and elevation in IOP of 2-3 mmHg [33], in our cases the temporal access phaco did not show any effect until the endpoint. The exiguous number of cases could explain this finding.

The sample size power was calculated for the IOP endpoint. Therefore, no conclusions can be drawn from the secondary endpoints, e.g. bleb morphology, number of postoperative medications and frequency of complications. We judge anyway that our results imply some interesting observations. Testifying to the persistence of the implant, the bleb height score was higher in the OLO than in MMC group at the 3rd month. Indeed, the residual implant volume added to the fluid-filled bleb spaces could justify this finding (since its biodegradation -according to the manufacturer- can last a 6-month period as above said). It is possible that a larger sample could have confirmed this difference in height for a longer period.

In studies using either morphologic grading scale evaluation or experimental 3D anterior segment SD-OCT, bleb height is one of the parameters correlated with a lower IOP [28, 34–36]. Whether at 24-months or 60-months follow-up, our sample shows that the outer bleb morphology by SD-OCT was quite similar between groups. These findings imply that outer layers of a functioning bleb are not modified by the OLO implant on a long term basis. In summary, the OCT bleb data indicate that bleb morphology is disappointingly not strongly correlated with function. A larger sample size could perhaps reveal that the SD-OCT pattern of a successful bleb, represents a prognostic factor for longer-term success. The end point bleb wall SD-OCT analysis confirms the critically reduced thickness of cystic thin blebs in both groups. The similarity of the whole bleb height in both groups, as measured by SD OCT, is in agreement with the above said clinical judgment by MBGS criteria.

The two groups did not differ with respect to adjunctive postoperative procedures. This could once more confirm similarity between the adjuvants.

Similar rates of early complications in the two groups further corroborate the equivalence of the effect of the two adjuvants during this postoperative period. The above said tendency to 1st day greater hypotony in MMC eyes did not reach significance in our sample. The low complication rate with low-dosage MMC within 2 years is in agreement with our previous studies [24, 37–39]. The cataract and AMD incidence could be ascribed both to aging and to glaucoma surgery in our cases. As previously noted, late thin avascular blebs are relatively common with MMC use. In our sample, after 5 years MMC cases exhibit a reduced vascularity together with a trend toward more frequent thin blebs, confirming the long-term effect from fibroblast mitosis inhibition [40]. It is surprising that, besides vascularity, in our sample there is no difference in end point bleb morphology of MMC cases when compared to the OLO ones, especially taking into account the diffuse area of application of MMC in the former and the limited surface of the 6x2 mm Ologen implant in the latter (see Fig. 3). The Ologen volume could hypothetically lift the surrounding conjunctiva from the scleral bed for a while, allowing a diffuse bleb, and/or, our sample size could imply some biased results, as above stated.

Earlier short to medium term retrospective or prospective randomized clinical trials denied our good results with Ologen. In fact, drawbacks such as lower bleb height and higher vascularity in OLO group, with a lower success rate, or higher complication rate of the Ologen-augmented trabeculectomy with respect to the simple one, or lower complete success rate even though a lower bleb-associated complication rate in Ologen vs MMC groups, are reported [10, 11, 41]. Variations in surgical procedure could justify these different outcomes. For instance, a non running suture with only two 10-0 nylon at the flap extremities likely lead to a higher rate of positive Seidel test with flat anterior chamber. Also, it could have reduced the role of the implant in bleb development in one study [10]. Additionally, the same study included 1-month topical steroid administration, vs 2 months with variable regimen based on IPC criteria in our study. This difference could have freed the vascular reaction in OLO eyes as aforementioned, resulting in an excess of fibroblasts surrounding the implant with higher end point IOP [10]. Finally, we note that small sample sizes, techniques of application and concentration of MMC, postoperative therapeutic regimen as well as variations in composition and different manufacturers of Ologen implants over the years could also be important factors to take into consideration [11, 12, 19, 41]. On the other hand, the difference between our results and those obtained by other centers in Germany, Singapore and India, could derive from the long-time known racial difference in the response to trabeculectomy, with white Caucasian performing better than Hispanic, East-Asian or Black population. The fibroblasts response to the Ologen implant could well follow this behavior [42, 43].

A one-year prospective interventional multicenter study including 30 eyes undergone trabeculectomy with MMC (0.2 mg/mL, 0.1 mL)-soaked Ologen, concludes that the procedure does not seem to exert any synergistic effect in terms of IOP reduction [20]. We note that, besides the lack of a control group, the MMC soaked Ologen can be by itself the cause of "ring of steel" bleb, due to the prolonged MMC localized effect with excessive surrounding fibroblast proliferation [23].

Conversely, the results of our study are in agreement, relating efficacy and safety, with recent shorter term studies comparing Ologen-augmented vs MMC-augmented trabeculectomy [14–18].

In particular, Johnson et al [17] in a 12-month retrospective review find that Ologen allows for similar success rates as MMC comparing respectively 49 vs 50 eyes of mainly POAG patients undergoing a filtering procedure using an Ex-PRESS (Alcon, Fort Worth, TX) mini glaucoma device.

A recent systematic review and meta-analysis indicates that trabeculectomy with Ologen is a safe and effective procedure in patients with glaucoma, and is comparable with MMC in IOP-lowering efficacy, even if does not seem to offer any significant advantages compared with trabeculectomy plus MMC [13].

Under this respect, especially considering the additional cost to the Ologen implant when compared to MMC, much larger trials are required to confirm possible advantages in terms of reduced incidence of early hypotony and late avascular-thin blebs. Once these requirements are met, the Ologen could be recommended as an alternative to MMC, at least in those cases where a target IOP in low teens is unnecessary. In the latter instance, an association between both adjuvants could be interesting.

## Conclusions

Our long-term study confirms that the OLO implant demonstrates efficacy in terms of IOP reduction, with a success rate quite similar to MMC, and at least equivalent safety. Moreover, outpatient surgery could benefit from this adjuvant if the lack of early significant hypotony will be confirmed by larger trials. Limits related to the small sample size must be overcome by further larger randomized trials to confirm the efficacy and safety of this device, and to accurately estimate any reduced risk for avascular and thin blebs with respect to MMC.

## Additional file

Additional file 1: Consort flow chart. (DOCX 48 kb)

## Abbreviations

OLO: Ologen®
MMC: mitomycin-C
VA: visual acuity
MD: Mean deviation
MBGS: Moorfields Bleb Grading System
SD-OCT: spectral domain optic coherence tomography
IOP: intraocular pressure
POAG: primary open angle glaucoma
PEXG: pseudoexfoliative glaucoma
IPC: intensified postoperative care
IOL: intraocular lens
SD: standard deviation
AMD: age-related macular degeneration
