# Supplementary material for: Biodegradable collagen matrix implant versus mitomycin-C in trabeculectomy: five-year follow-up
Source: BMC Ophthalmol. 2016 Mar 5;16:24. doi: 10.1186/s12886-016-0198-0 (PMC4779569; doi:10.1186/s12886-016-0198-0)
Supplement: Additional file 1: — Consort flow chart. (DOCX 48 kb) [file 12886_2016_198_MOESM1_ESM.docx]

Excluded (n=0)

## Enrollment

Randomized (n=40)

## Allocation

Allocated to trabeculectomy with MMC (n=20)

♦ Received trabeculectomy with MMC (n= 20)

Allocated to trabeculectomy with Ologen (n=20)

♦ Received trabeculectomy with Ologen (n= 20)

## Follow-Up

Analysed (n=20)
♦ Excluded from analysis (n=0)

Analysed (n=20)
♦ Excluded from analysis (n=0)

## Analysis

Lost to follow-up (n=0)

Discontinued intervention (n=0)

Developed cataract (n=2)

♦ underwent phaco with IOL (n= 2)

Lost to follow-up (n=0)

Discontinued intervention (n=0)

Developed cataract (n=3)

♦ Underwent phaco with IOL (n= 3)

Assessed for eligibility 40 patients (40 eyes)
